# EGFRvIII-specific CAR-T cells produced by piggyBac transposon exhibit efficient growth suppression against hepatocellular carcinoma

**DOI:** 10.7150/ijms.45603

**Published:** 2020-06-05

**Authors:** Yuan Ma, Yan Chen, Lei Yan, Hui-Xia Cao, Shuang-Yin Han, Jun-Jun Cui, Jian Guo Wen, Yan Zheng

**Affiliations:** 1 Key Laboratory of Clinical Medicine Henan Universities, The First Affiliated Hospital of Zhengzhou University, Zhengzhou, 450003, China.; 2 Key Laboratory of Immunology and Kidney Disease, People's Hospital of Henan Province, Zhengzhou University, Zhengzhou, 450003, China.; 3 Physical Examination Department, People's Hospital of Zhengzhou, Zhengzhou, 450003, China.

**Keywords:** EGFRvIII, chimeric antigen receptor, hepatocellular carcinoma

## Abstract

Adoptive cellular immunotherapy employing chimeric antigen receptors-modified T (CAR-T) cells has demonstrated promising antitumor effects in hematologic cancers. However, CAR-T therapy confront many challenges in solid tumors like immunosuppressive microenvironment, molecular heterogeneity, etc. The cancer genome atlas (TCGA) of hepatocellular carcinoma (HCC) revealed many genetic characteristic and molecular tumorigenesis. EGFRvIII is a tumor specific antigen widely expressed in a variety of cancers including HCC and an ideal therapeutic target for cancer therapy. The liver cancer cell line SMMC7721 express high level EGFRvIII and widely applied in HCC investigations. Herein, we developed EGFRvIII CAR-T cells by piggyBac transposon system, and detected its specific killing effect against SMMC7721 cells *in vitro* and *in vivo*. Results indicated that transduction efficiency of CAR reached 53.1%. Expression of CAR protein was verified by immunoblotting as a band of approximate 57KD. The killing effect of CAR-T cells against SMMC7721 was positively correlated with E/T ratio (E:T=5:1, 10:1, 20:1, 40:1), and exceeded 50% at 20:1 ratio. Significant increase in IFN-γ and TNF-α secretion were detected in the co-culture supernatant of CAR-T cells and SMMC7721, comparable to the level of exogenous EGFRvIII-expressing U87 cells. The killing activity and cytokine secretion were both dependent on the expression level of EGFRvIII in target cells. In HCC xenograft models, CAR-T cells could effectively suppress the growth of SMMC7721. In conclusion, EGFRvIII CAR-T cells demonstrated specific antitumor effect against SMMC7721 *in vitro* and *in vivo*, providing basis for immunotherapy of HCC in future clinical use.

## Introduction

Adoptive transfer of chimeric antigen receptor-engineered T (CAR-T) cells is an attractive strategy for tumor immunotherapy 1. CAR combines an antigen-binding domain with a CD3ζ signaling motif into a single chimeric protein to redirect the specificity of T cells in an MHC-independent fashion. Use of CAR-T cells as a treatment for cancer has been extensively investigated, and achieved remarkable outcomes in patients with B cell malignancies 2. In parallel with the clinical trials in hematological malignancies, CAR-based therapy has also been conducted in solid tumors. However, the results of pilot clinical trials on solid cancers are below expectation. Moreover, rapid death caused by the off-tumor cross-reaction of CAR-T cells has been reported 3. Molecular target selection is the precondition for the implementation of CAR-T cell therapy, which not only related to the specificity/effectiveness, but also correlated to the toxicities 4,5. Suitable tumor specific/associated antigen (TSA/TAA) is probably one of critical determinant to successful CAR therapy.

Epidermal growth factor receptor variant III (EGFRvIII) is the most common variant of the EGF receptor. It results from the in-frame deletion of exons 2 to 7 and the generation of a novel glycine residue at the junction of exons 1 and 8. This novel juxtaposition of amino acids within the extracellular domain of the EGFR creates a tumor-specific, oncogenic, and immunogenic epitope 6. This variant receptor expressed in numerous types of human tumors such as glioblastoma, breast, non-small-cell lung, liver and ovarian cancers 7. The frequency of EGFRvIII expression on HCC is 30-60%. Its expression is closely correlated with the malignant phenotype 8,9. SMMC-7721 is liver cancer cell lines generated from Chinese 50-year-old male HCC patient 10. EGFRvIII expression on SMMC-7721 is well studied and widely used as a target cells for cancer immunotherapy 11,12. In this study, we developed the second-generation of EGFRvIII targeted CAR-T cells based on the piggyBac transposon system. The genetically engineered T cells demonstrated a specific and efficient cytotoxicity against EGFRvIII expressing liver cancer cell *in vitro* and *in vivo*. Given that EGFRvIII is expressed in HCC, we explored the potential application of EGFRvIII CAR-T cells as a novel intervention for this deadly disease.

## Materials and methods

### Cell lines

Human liver cancer cell line SMMC-7721 (with endogenous stable expression of EGFRvIII) and human glioma cell line U87 were purchased from Chinese Academy of Science in Shanghai and maintained in Dulbecco's modified Eagle's medium (DMEM, Hyclone) supplemented with 10% FBS. Stable EGFRvIII expressing U87 cell line (EGFRvIII-U87) was generated by our lab and cultured in DMEM containing 10% FBS and puromycin (5ug/ml). Cells were cultivated at 37 °C and 5 % CO_2_ in a humidified incubator.

### CAR construct and plasmids

EGFRvIII CAR is composed of EGFRvIII scFv and CD137-CD3ζ expression cassette. The EGFRvIII scFv was derived from a high-affinity EGFRvIII monoclonal antibody described previously with the order of a light chain-(GGGS)_3_-a heavy chain (726bp). The affinity of this antibody for EGFRvIII peptide is 1.7×10^7^ M^-1^ 13. The piggyBac transposon and transposase plasmids (PB513B-1, PB200PA-1) were purchased from System Biosciences and purified by standard techniques using EndoFree kits (Invitrogen). EGFRvIII CAR was cloned into transposon vector via EcoRI/XbaI restriction endonuclerase sites. The sequence of EGFRvIII CAR was confirmed using DNA sequencing analysis.

### Generation of EGFRvIII CAR-T cells

Peripheral blood mononuclear cells (PBMCs) from healthy donors were isolated by Ficoll-Hypaque gradient centrifugation (Solarbio) and monocytes were removed by adherent plastics. Then washing obtained T cells with phosphate-buffered saline (PBS) twice. About 10^7^ cells were counted and suspended with CAR-transposon (5ug) and transposase (5ug) plasmid in 120μl electroporation buffer. The electroporation condition (840V, 20ms) was used according to the instructions. After electroporation, all cells were activated utilizing human recombinant EGFRvIII antigen/anti-CD28 antibody (EGFRvIII/CD28, 5μg/mL) coated plates for 2-3 days, and then placed in new 6-well plates for expansion. T cells after electroporation were cultured in X-VIVO 15 medium (Lonza) containing 100IU/mL IL-2, 10ng/mL IL-15 and 10ng/mL IL-7 (Peprotech). Fresh medium and cytokines were added every other day until day 7.

### EGFRvIII CAR-T cells transgene expression and viability

Twenty-four hours post-electroporation, the percentage of GFP-positive cells was measured by flow cytometry to detect transfection efficiency of foreign gene. Cell viability was detected by apoptosis detection kit (Biolegend). Cells were stained with Annexin V-APC and 7-aminoactinomycin D (7-AAD) stain, to discriminate intact cells (Annexin-/7-AAD-) from apoptotic cells (Annexin+), and necrotic cells (Annexin-/7-AAD+). Cell mortality was also calculated by Cellometer Auto2000 (Nexcelom Biosciences). To evaluate long-term transgene expression, the percentage of fluorescent cells were identified at day 1, 3, 5 and 7 in the usual manner by automated cytometer (Cellometer Auto 2000) as previously described 14.

### CCK8 proliferation assay

To examine whether CAR-T cells can be specifically activated by tumor antigen, CCK8 proliferation assay was performed. T cells were counted and transferred to EGFRvIII/CD28 coated 96-well plates with 1.5×10^4^ cells/well for five replicates. CCK8 (Dojindo) was added to each well at 24h, 48h, 72h, and then incubated for 5h. OD_450_ values were achieved by Synergy H1 Hybrid Reader (BioTek) after incubation and analyzed with Gen5 software.

### Flow cytometry

To detect phenotype and subset, CAR-T cells were stained with mouse anti-human CD4 Percp, CD8 PE, CD62L PE, CCR7 Percp and CD45RO PE/Cy7 on day 7 (Biolegend). To determine CAR transgene, Alexa 647-labeled F(ab)_2_ fragment of Goat anti-Mouse IgG (Jackson ImmunoResearch) was utilized. All samples were analyzed with canto II (BD Biosciences), and data were processed by FlowJo software.

### Western blotting

To confirm the expression of EGFRvIII CAR protein, T cells (3×10^6^) were lysed in 100μl lysis buffer. After centrifugation, cell lysates were run on 12% SDS-PAGE under reducing conditions and transferred to PVDF membrane (Millipore) for immunoblot analysis. Membranes were incubated with mouse anti-human CD3ζ (Santa Cruz). Moreover, EGFRvIII expression in target cell was also detected. The samples were immuno-blotted with mouse anti-human EGFRvIII (Novus). Horseradish peroxidase conjugated anti-mouse IgG (Santa Cruz) was used as the secondary antibodies. Blots were visualized using the ECL (Millipore).

### *In vitro* cytotoxicity and cytokine release assays

To determine if EGFRvIII CAR-T cells were able to lyse the target cell SMMC7721, cytotoxicity measurements were performed using CytoTox96® Non-Radioactive Cytotoxicity kit (Promega) based on LDH release. SMMC7721 cells were mixed with increasing numbers of effector cells at effector-to-target ratios of 5:1, 10:1, 20:1 and 40:1. To measure cytokine production, effector cells were co-incubation with target SMMC7721 cells at E:T=20:1 ratio. Culture supernatants were harvested after 12 hours and the production of IFN-γ and TNF-α was determined by ELISA (R&D). EGFRvIII-U87, U87 cells were used as positive and negative control, respectively.

### *In vivo* antitumor activity of EGFRvIII CAR-T cells

Xenograft tumor model was established by subcutaneous flank injections of 1×10^7^SMMC7721 cells in 6-week-old female BALB/cA-nude mice (Chinese Academy of Science Shanghai Experimental Animal Center). When the tumor burden reached about 500 mm^3^ at day 14 after tumor cells inoculation, the mice were assigned to different groups (4 in each group) and injected with 1×10^7^ different T cells/100μl (EGFRvIII CAR-transduced T cells, non-transduced T cells, and control PBS) systemically to tail vein. Tumor growth was subsequently monitored by caliper measurement and tumor volume was calculated using the formula: 1/2 × length × (width)^2^ . The mice were killed when tumor volume reached >1500 mm^3^. Immunohistochemistry (IHC) was performed to examine the expression of EGFRvIII in the treated tumor tissues with rabbit anti-EGFRvIII antibody (Bioss) as previously described 15.

### Statistical analysis

All statistical analyses in this study were performed with SPSS (version 20.0). Data were analyzed by Student's t test when comparing two sets of data, or ANOVA for multiple comparisons. P-values less than 0.05 were considered statistically significant. Data was presented as mean ± standard deviation.

## Results

### EGFRvIII CAR-T cells can be successfully generated by piggyBac transposon system

**Figure 1:**
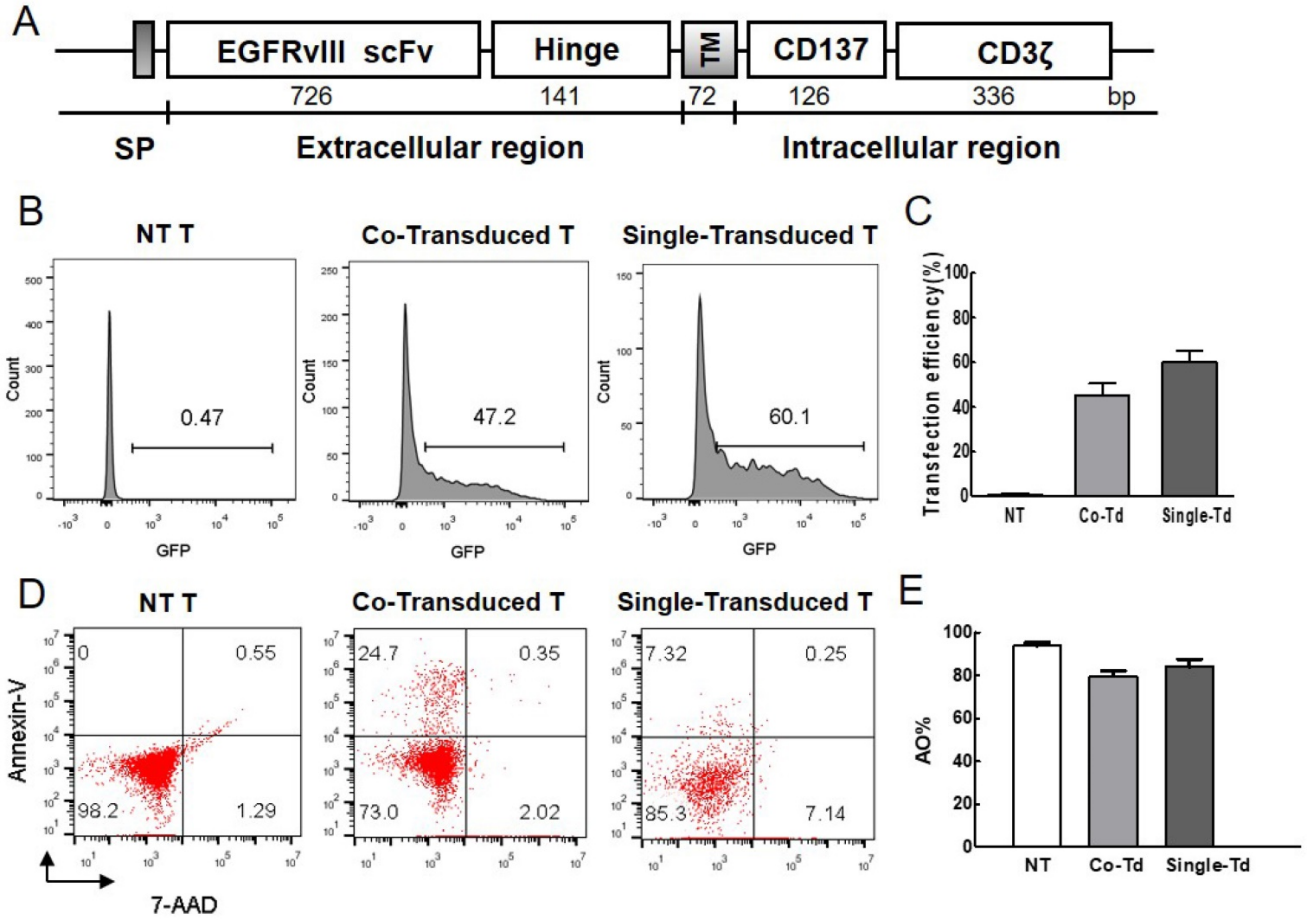
** Evaluation of transfection efficiency and viability. (A)** Structure of EGFRvIII CAR. It contained EGFRvIII scFv, the hinge and transmenbrane (TM) region of human CD8α, CD137 signaling domain, and human CD3ζ chain. IgG κ chain was used as signal peptide (SP).** (B)** The percentage of GFP-positive cells represented transfection efficiency of foreign gene at 24h after electroporation. Non-transduced cells were used as control. **(C)** Transfection efficiency was detected by flow cytometry (n=3). **(D)** Cell mortality post-electroporation was examined by flow cytometric analysis. Cells were stained with Annexin V-APC and 7-AAD dye, the fraction of Annexin^+^/7-AAD^-^ dead cells was indicated. **(E)** AO/PI dual-fluorescence for live/dead staining was also used to quantify cell survival rate and detected by automated cytometer (n=3). Co-Transduced T (Co-Td): T cells was transfected with CAR-transposon and transpoase. Single-Transduced T (Single-Td): T cells was transfected with CAR-transposon. NT T: non-transduced T cells.

A second generation of EGFRvIII CAR containing the EGFRvIII scFv, CD137 signaling domain and CD3ζ chain was constructed, and located within the piggyBac transposon cassette (Figure 1A). Transfection efficiency of foreign gene was detected by flow cytometer. More than 40% of T cells obtained GFP expression without selection (Figure 1B, C) on day 1 post-electroporation. Less than 25% of T cells were dead (Figure 1D, E). The result showed that T cells can obtain efficient gene transfer with low mortality rate.

**Figure 2:**
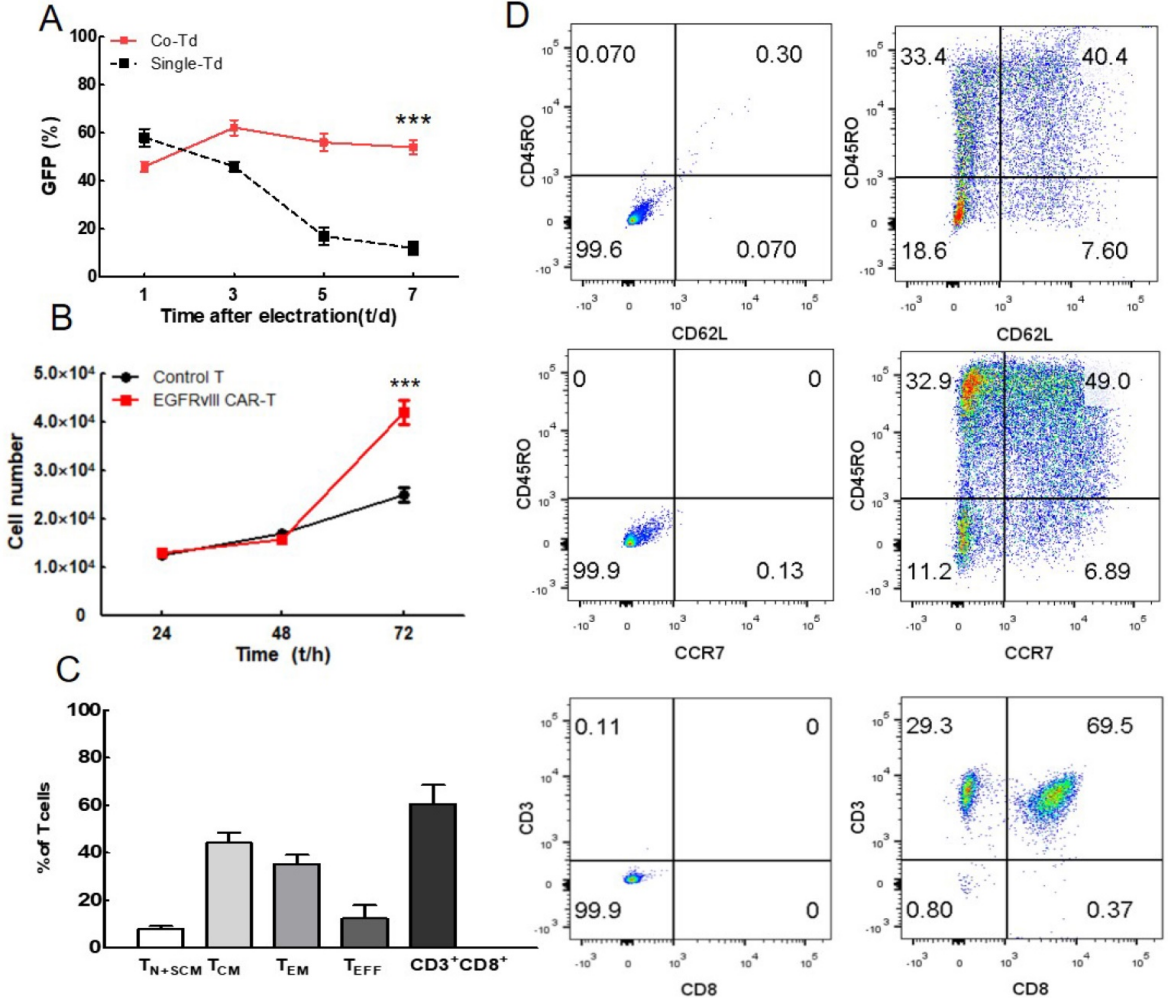
** Analysis of CAR-T cells activation, proliferation and phenotype. (A)** Change in the percentage of positively transfected cells after electroporation (n=4). **(B)** Proliferation of EGFRvIII CAR-T cells and non-transduced T cells were assessed by CCK8 assays (n=3). Cell numbers were calculated via standard curves based on OD_450_ values. **(C)** T cell phenotype and subset composition were analyzed by flow cytometry on day 7 (n=3), Non-transduced T cells were used as control. **(D)** A representative cell phenotype analysis. ^***^P <0.001.

To observe the transposition effect of transposase, we analyzed the GFP positive cells at day 1, 3, 5 and 7 as shown in Figure 2A. The CAR-transposon plasmid with or without transposase plasmids were electroporated into T lymphocytes. Compared with single-transduced lymphocytes, the co-transduced lymphocytes exhibited higher levels of GFP expression at the end of the culture (mean 54% vs 12%). These results showed that foreign gene can be transfected into T lymphocytes in the absence of transposase, but not integrated in T cell genome. Transposable elements played an important role in PB transposition. In addition, persistent expression of exogenous gene can be achieved in co-transduced lymphocytes activated by EGFRvIII/CD28. The representative expansion procedure was shown by fluorescence microscope in supplemental material (Figure S1).

CCK8 proliferation assay was performed to further verify the specific stimulation of CAR-T cells using EGFRvIII antigen (Figure 2B). When activated by EGFRvIII/CD28, EGFRvIII CAR-T cells proliferated more significantly than non-transduced T lymphocytes, indicating that EGFRvIII antigen can specifically induce EGFRvIII CAR-T activation.

### Expanded CAR-T cells exhibited less-differential phenotype

CAR-T cells were then cultured in the presence of IL-2, IL-7 and IL-15, and immune phenotype was detected by flow cytometry on day 7 (Figure 2C, D). Less-differentiated cells consisted of T_N_, T_SCM_ and T_CM_ accounted for (52.2±4.0) % of T cells. This cell population is critical for *in vivo* expansion, survival, and long-term persistence. Besides, a large majority of the cultured cells were CD3^+^CD8^+^ T cell subset (60.6±8.1) %, which was highly related to the cytotoxicity of T cell.

### EGFRvIII CAR-T cells exerted specific cytotoxicity against SMMC7721 cells *in vitro*

**Figure 3:**
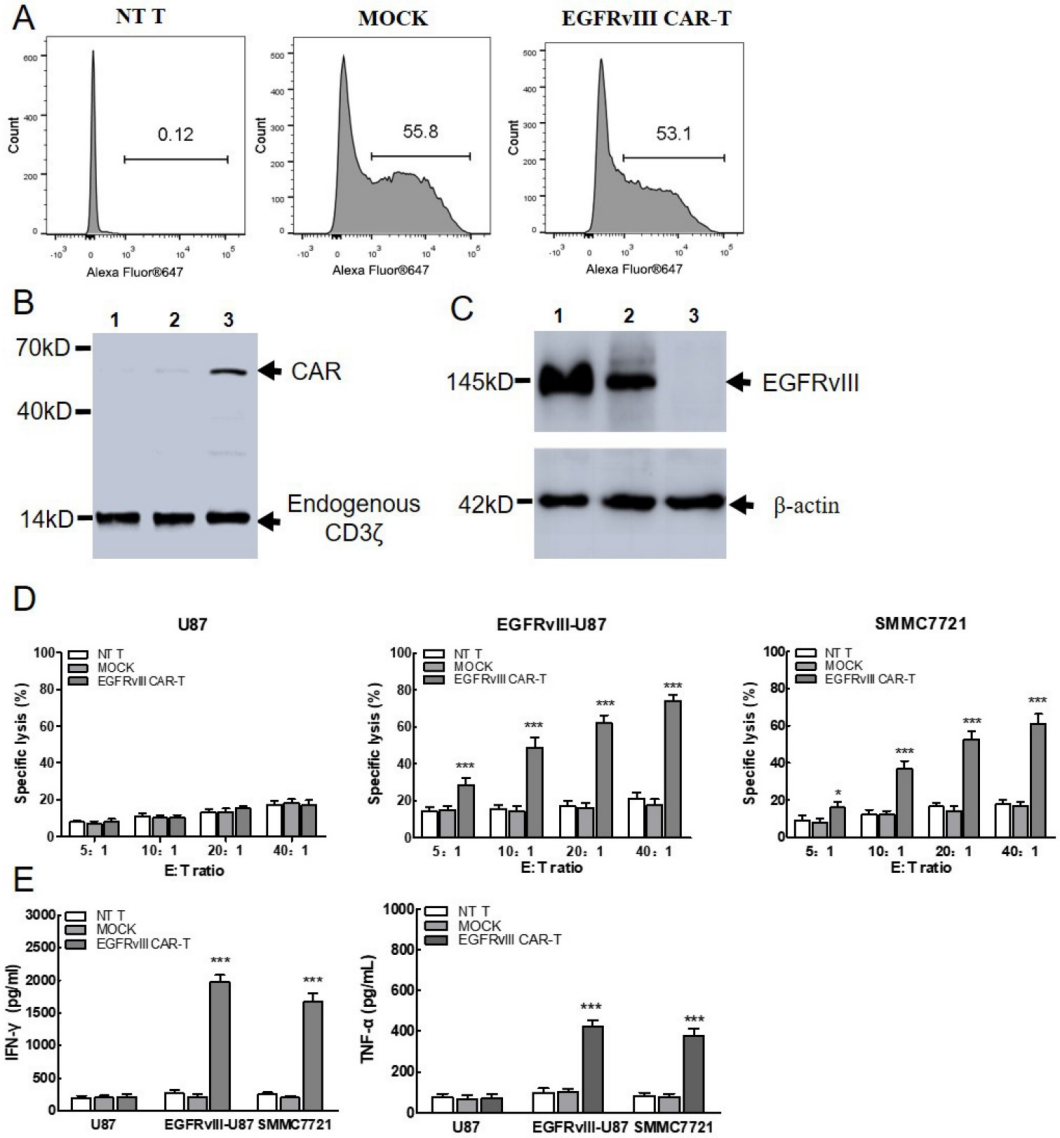
***In vitro* functional analysis of EGFRvIII CAR-T cells. (A)** Representative flow cytometry analysis of transduction efficiency of EGFRvIII CAR. Surface expression of CAR on T cells was detected by anti-mouse F(ab)_2_-Alexa 647. NT T and MOCK were used as control. NT T: Non-transduced T cells MOCK: T cells expressing CARs that encode the truncated CD3ζ. **(B)** Immunoblot analysis of CAR expression. Lysates were separated by SDS-PAGE under reducing condition. Mouse anti-human CD3ζ antibody was used to detect the endogenous and chimeric CD3ζ expression. 1: NT T cells, 2: MOCK, 3: EGFRvIII CAR transduced T cells. **(C)** Immunoblot analysis of EGFRvIII expression in target cells. 1:EGFRvIII-U87 cells, 2:SMMC7721, 3:U87 cells. **(D)**When co-incubated with SMMC7721 cells, EGFRvIII CAR-T cells cytotoxicity was measured by Non-Radioactive Cytotoxicity kit. CAR-T cells which were co-cultured with EGFRvIII-U87 or U87 cells were used as positive or negative control, respectively (n=3). **(E)** Cytokine production of CAR-T cells was quantified by ELISA. EGFRvIII-U87 and U87 cells were used as positive and negative target cell control (n=3). ^***^P <0.001,^*^P <0.05.

The surface expression of EGFRvIII CAR on T cells was detected by flow cytometry using anti-mouse IgG F(ab)_2_-Alexa 647. As shown in Figure 3A, CAR expression efficiency reached 53.1%, which was sufficient for the further experiment. Then, the expression of EGFRvIII CAR protein was confirmed by Western bolt (Figure 3B). Endogenous CD3ζ chain was detected as a 14kD band in both lanes. Band of approximate 57KD was detected as CAR in the T lane, but not in non-transduced T cells and MOCK. Results showed that EGFRvIII CAR was successfully expressed on the surface of T lymphocytes. In addition, EGFRvIII expression in target cell was also detected in Figure 3C. Protein expression was detected as a 145KD band in SMMC7721 cells (lane 2), which was consistent with that in the positive control EGFRvIII-U87 cells (lane 1), but not in the negative control U87 cells (lane 3). Results indicated that SMMC7721 cells can express endogenous EGFRvⅢ.

To determine whether the EGFRvIII CAR-T cells can recognize and kill the SMMC7721 target cells, a cytotoxicity detection assay based on LDH release was performed (Figure 3D). After co-cultured with SMMC7721 overnight, EGFRvIII CAR-T cells killing effect was detected at 5:1, 10:1, 20:1, 40:1 (E:T). A robust enhancement in the cytotoxicity was detected as an increase in E:T ratio. The killing activity exceeded 50% as E:T ratio increased to 20:1, comparable to the level of positive control target cells (EGFRvIII-U87) group. In addition, a significant difference was noticed in the EGFRvIII expressing target cells at each E:T ratio between EGFRvIII CAR-T cells and MOCK or NT T cells. In contrast, no evident killing activity was found among T cells of three different groups toward EGFRvIII-negative U87 cells. These results confirmed the specificity and efficiency of cytotoxic T cell response against SMMC7721 cells when EGFRvIII CAR was grafted onto T cells.

### IFN-γ and TNF-α secretion of EGFRvIII CAR-T cells was increased in SMMC7721 cells

To determine whether EGFRvIII CAR-T cells become activated and acquire effector cell functions when encountering SMMC7721, cytokine release assay was utilized (Figure 3E). After co-cultured with SMMC7721 overnight, EGFRvIII CAR-T cells released (1677±125) pg/ml IFN-γ and (378±32) pg/ml TNF-α at E:T=20:1, which was similar with positive control target cells (EGFRvIII-U87). In contrast, cytokine release remained unchanged in control effector cells (NT T cells and MOCK) or negative control target cells (U87 cells).

### EGFRvIII CAR-T cells can effectively inhibit tumor growth *in vivo*

**Figure 4:**
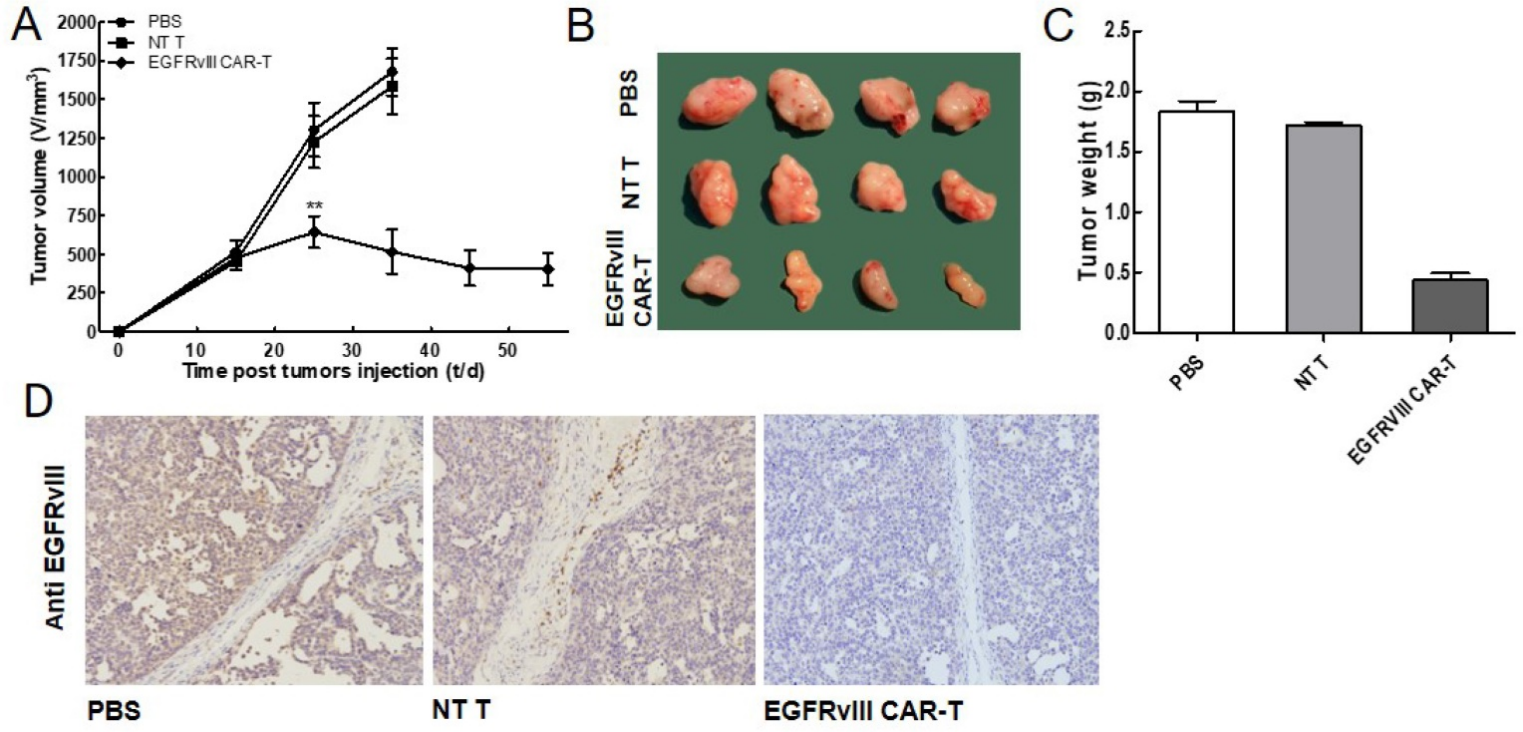
***In vivo* EGFRvIII CAR-T cells antitumor activity. (A-C)** The SMMC7721 cells were used for establishing xenograft mouse model. The tumor volumes and weights were quantified. EGFRvIII CAR-T significantly inhibited the growth of SMMC7721 tumors, compared with control group (PBS or NT T cells) (n=4). **(D)** Tumors were collected from mice with SMMC7721 xenografts treated with EGFRvIII CAR-T cells, PBS or NT T cells. Paraformaldehyde-fixed (4%), paraffin-embedded tumor sections were consecutively cut and stained for EGFRvIII expression (brown). Images were obtained with a microscope (Olympus) under×200 magnification. ^**^P <0.01.

To further evaluate whether EGFRvIII CAR-T cells inhibited tumor growth *in vivo*, a xenograft model by inoculating the SMMC7721 in the flanks of nude mice was developed. The tumor-loaded mice were received injections of 1×10^7^ EGFRvIII CAR-T cells, NT T cells and PBS, respectively. Tumor volume in mice receiving EGFRvIII CAR-T cells started to shrink four weeks after adoptive cell transfer, while tumors in other two groups continued to grow (Figure 4A, B). The values of the tumor size were concordant with those of the tumor weights (Figure 4C). We excluded the possibility that the antitumor effect of the EGFRvIII CAR^-^T cells resulted from their allogeneic effect because the inoculated NT T cells did not show any effects on tumor growth. Next, we examined EGFRvIII expression in the treated tumor sections by IHC analysis (Figure 4D). The IHC results showed that there were fewer EGFRvIII-positive tumor cells in the group of EGFRvIII CAR-T cells compared with the control groups (treatment with NT T cells or PBS alone). The results of these experiments demonstrated that the EGFRvIII CAR-T cells could effectively suppress the growth of SMMC7721 cells *in vivo*.

## Discussion

Genetic engineering of T cells to express chimeric antigen receptors (CARs) is a promising new approach for adoptive cell therapy. CARs are chimeric proteins combine an antigen recognition domain of a specific antibody with an intracellular domain of the CD3ζ chain. Clinical trials testing CARs are presently under way at a number of academic medical centers. The most successful clinical results of CAR-T treatment were achieved in hematological cancer 16, 17. Based on the success in hematological malignancies with CAR-T cells, there is now mounting interest around how to use CAR-T cells for the treatment of solid tumors. However, the application of CAR-T cells to solid tumors has been limited by several obstacles, lacking of tumor specific antigen included.

Hepatocellular carcinoma (HCC) is the third-most lethal cancer worldwide. Less than 30% of HCCs are diagnosed at an early stage and are amenable for resection, liver transplantation, or local ablation 18. Despite advances in treatment, the five-year survival rate of patients with HCC remains poor averaging 5-15% 19. Finding an effective therapeutic for HCC remains an unmet need. Recently, CAR-T cells targeted HCC-associated antigen GPC3 have achieved promising clinical trials outcomes 20. Another target antigen EGFRvIII, for its tumor specific and oncogenic property, has become an increasingly attractive molecule for tumor immunotherapy. Its expression was also detected in HCC tissue samples, closely correlates with increased tumorigenicity. SMMC7721, as a hepatocarcinoma cell line, is established from a Chinese HCC patient and is commonly used in the research of HCC at home and abroad. Anticancer effects and mechanisms of different anti-cancer drugs have been experimented on SMMC-7721 cell line. EGFRvIII molecule is endogenous stably expressed on SMMC7721, which makes it suitable to evaluate the targeted and killing efficiency of CAR-T cells for HCC immunotherapy.

In the present study, we developed a second generation of EGFRvIII targeted CAR-T cells. EGFRvIII-U87 target cells were utilized as positive control to verify the efficacy of EGFRvIII CAR-T cells *in vitro* and vivo. When co-cultured with SMMC7721, these redirected T cells were demonstrated to exert efficient cytotoxic T cell response on SMMC-7721 cells in an EGFRvIII specific manner. Killing activity exceed 50% at the E:T ratio of 20:1. In addition, the enhanced secretion of cytokine IFN-γ and TNF-α illustrated the activation of CAR-T cells by SMMC7721 cells, which is performed in an antigen dependent way. Importantly, EGFRvIII CAR-T cells showed effective anti-tumor effect on SMMC-7721 xenografts. The nude mouse loaded tumor in the CAR-T treated group still survived for more than 2 months, while mouse in the control group were sacrificed due to rapid growth of the tumor.

Apart from a careful choice of the target antigen, an easier and safer CAR-T manufacture method is indispensable for CAR-based immunotherapy. Current manufacture of clinical-grade CAR T cells is primarily based on lentiviral/retroviral (LV/RV) transduction of T cells, leading to an average of 30% to 80% transduction efficiency. However, the manufacturing process is labor-intensive and complex processes. As an alternative, PB transposon system-based non-viral transduction method has been considered to circumvent some of these limitations. For most applications, the piggyBac transposon and transposase are carried on two separate plasmids (trans), and gene transfer is carried out through a 'cut and paste' mechanism 21. Herein, we developed the EGFRvIII modified CAR-T cells by piggyBac transposon system, which is a faster and more economical method. EGFRvIII CAR-T cells were activated by EGFRvIII antigen, and transfection efficiency was mean 54% on day 7, at a level comparable to that achieved by the viral transduction. Cell mortality was less than 25%, which is comparable to the widely used nucleofector technique 22.

After successfully establishing the primary T lymphocyte transduction technology platform, we evaluated cell phenotype and composition. It becomes increasingly clear that adoptive transfer of the less-differentiated T cell subsets is associated with superior T cell engraftment, persistence, and antitumor immunity. In the current study, apart from conventional IL-2, IL-7 and IL-15 was also added in the cultivation to promote the generation of young T cells 23, 24. More than 50% of CAR-T cells exhibited a less-differentiated phenotype. Overall, EGFRvIII CAR-T cells can be successfully generated by piggyBac transposon system, and exhibited specific and effective cytotoxicity against EGFRvIII expressing target cells *in vitro* and vivo, which may be a promising option for HCC therapy.

## Supplementary Material

Supplementary figures and tables.
