# Supplementary material for: EGFRvIII-specific CAR-T cells produced by piggyBac transposon exhibit efficient growth suppression against hepatocellular carcinoma
Source: Int J Med Sci. 2020 Jun 5;17(10):1406–14. doi: 10.7150/ijms.45603 (PMC7330669; doi:10.7150/ijms.45603)
Supplement: Supplementary file 1 — Supplementary figures and tables. [file ijmsv17p1406s1.pdf]

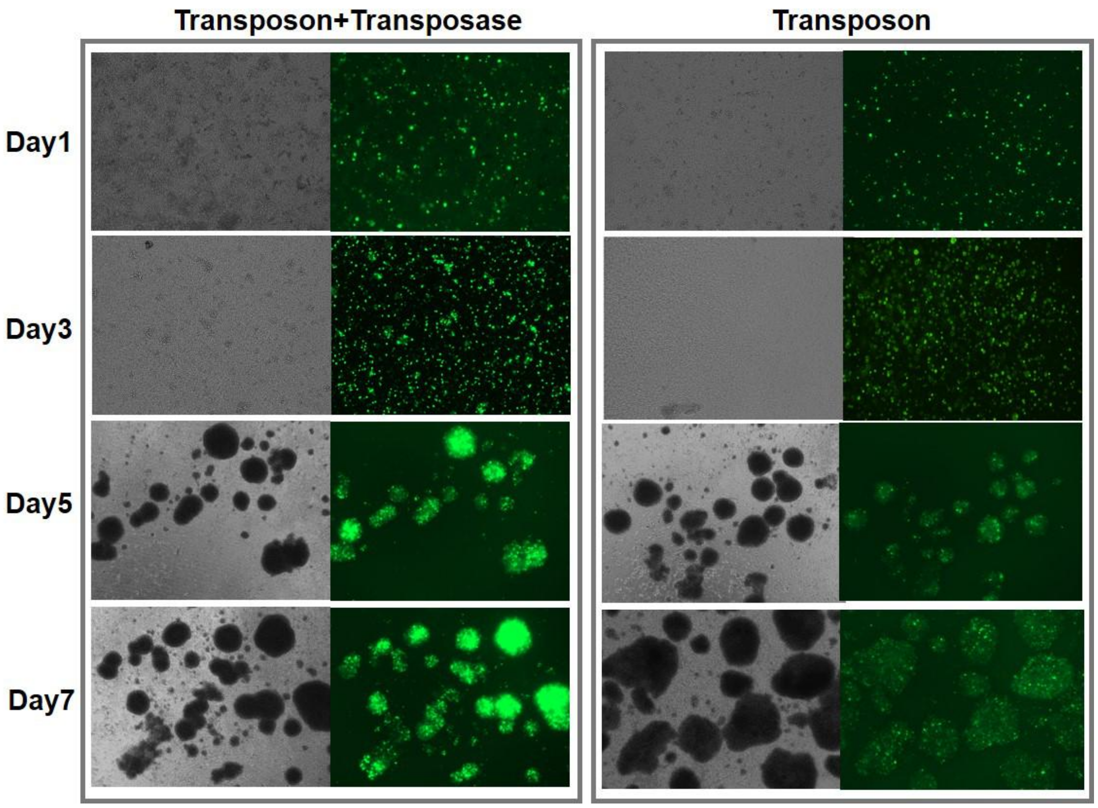

Figure S1. GFP expression was recorded by fluorescence microscope at day 1, 3, 5 and 7. The expression efficiency of foreign gene was gradually increased as electrotransfected with both transposase and transposon, while the opposite without transposase.
